# Genetic evaluation of productive longevity in a multibreed beef cattle population

**DOI:** 10.1093/jas/skae363

**Published:** 2024-11-29

**Authors:** Tiago Luciano Passafaro, Yeni Liliana Bernal Rubio, Natascha Vukasinovic, Dianelys Gonzalez-Peña, Daniel Gustavo Mansan Gordo, Thomas Short, Lee Leachman, Kent Andersen

**Affiliations:** Zoetis Inc, Livestock Genetics and Precision Animal Health VMRD, Kalamazoo, MI 49007, USA; Zoetis Inc, Livestock Genetics and Precision Animal Health VMRD, Kalamazoo, MI 49007, USA; Zoetis Inc, Livestock Genetics and Precision Animal Health VMRD, Kalamazoo, MI 49007, USA; Zoetis Inc, Livestock Genetics and Precision Animal Health VMRD, Kalamazoo, MI 49007, USA; Zoetis Inc, Livestock Genetics and Precision Animal Health VMRD, Kalamazoo, MI 49007, USA; Zoetis Inc, Livestock Genetics and Precision Animal Health VMRD, Kalamazoo, MI 49007, USA; Leachman Cattle of Colorado, Fort Collins, CO 80524, USA; Zoetis Inc, Livestock Genetics and Precision Animal Health VMRD, Kalamazoo, MI 49007, USA

**Keywords:** beef cattle, multibreed, number of calves, productive longevity, repeatability

## Abstract

Genetic selection for traits that have a direct impact on profitability, such as productive longevity (**PL**), which blends cow longevity with regular reproductive performance, is fundamental for the economic success of beef cow-calf operations. The purpose of this study was to develop a data screening strategy and a statistical model to predict genetic merit for PL in a multibreed beef cattle population. Pedigree (*n* = 1,352,765) and phenotype (*n* = 978,382) information were provided by Leachman Cattle of Colorado, and genotypes (*n* = 26,342) were provided by the Zoetis commercial genotyping laboratory. A repeatability model (**REP**) including the systematic effects of age at first calving, year-season of progeny birth, pedigree-based retained heterosis, and parity number, as well as the random effects of the additive genetic, permanent environment, contemporary group, and residual were fitted to adjust PL. In addition, a random regression model (**RRM**) was fitted to investigate PL considering the same effects, with the difference that random effects were regressed on parity. Estimated breeding values (**EBV**) were obtained by single-step GBLUP (**ssGBLUP**) and transformed to predict differences in the number of calves through linear regression. Predictive performance was assessed in a group of 7,268 cows born in 2010. Heritability estimates for PL were relatively low, with values of 0.109 for REP and a decreasing trend for RRM with values ranging from 0.16 to 0.04. Repeatability for PL was of moderate magnitude, with values of 0.415 for REP and from 0.29 to 0.57 for RRM. Heritability estimates suggest that most of the phenotypic variation was accounted for by environmental factors, but long-term genetic selection could still be effective. REP was more efficient than RRM, showing the lower number of iterations and time to reach convergence with comparable solutions to RRM. Validation results showed that correlations between EBV and phenotypes (observed/precorrected) increased over the years ranging from 0.04 to 0.92. Repeatability values and the validation approach suggested that using a cow’s first record (second parity success or failure) is a reasonably good indicator of posterior performance for PL. Therefore, the inclusion of PL in a multibreed genetic evaluation program and incorporation into selection indexes with existing economic traits can enable more profitable selection and breeding decisions in beef cattle herds.

## Teaser

This paper presents a concept called productive longevity (**PL**) that blends cow longevity with reproductive performance measured by the number of calves regularly produced in the herd. Identifying animals with high genetic merit for PL is crucial to inform selection and breeding decisions, assisting beef cattle producers to improve profitability.

## Introduction

Profitability is a key component of the long-term sustainability of beef cattle production around the globe. Investing in genetic evaluation and selection of economically relevant traits and indicators that directly impact profitability is crucial for the overall success of beef cow-calf operations. In this context, cow longevity constitutes one of the main profitability drivers in beef cow-calf operations by decreasing costs related to cow replacement and rearing heifers and the sale of offspring with heavier weights (Garrick, 2006). Santos et al. (2019) indicated that improving longevity was the utmost priority by beef cattle producers in a survey developed in the United States.

Longevity is often defined as the life period of a cow after the first calving to the natural death (Olechnowicz et al., 2016; Zhang et al., 2016). Such a definition accounts for the duration of life but has no consideration on cow’s productive performance. Therefore, alternative definitions of longevity that blend longer lifespan and reproductive performance were developed to help maximize the profitability of beef cattle operations. Martinez et al. (2005) proposed longevity as a measure of the probability of a cow staying in the herd up to a specific age given that she calved at 2 yr of age. Jamrozik et al. (2013) proposed longevity in terms of consecutive calving, while Venot et al. (2013) defined it as the number of calves produced up to a target age. Regardless of the definition used for longevity, all approaches have advantages and disadvantages. For instance, the traditional definition of stayability computes the probability of a cow remaining in the herd until a specific age, given she calved once, without taking into consideration the total number of calves produced over lifetime. The definition proposed by Jamrozik et al. (2013) has the advantages of considering multiple records per cow and allowing the inclusion of younger animals in the evaluation with censored data, which helps increase accuracy for younger bulls. However, in the study of Jamrozik et al. (2013), a linear model was applied to a binary phenotype, which has the drawback of reporting genetic merit values with no units, making interpretation challenging. Another disadvantage of this definition is to deal with binary phenotypes that are relatively more challenging to adjust a model compared with continuous traits. Furthermore, the definition proposed by Venot et al. (2013) has the benefit of taking into consideration cow’s reproductive performance, but with the caveat of having a longer generation interval as cows need to reach a specific age to be included in the evaluation. With that in mind, we are proposing an algorithm to investigate longevity inspired by Venot et al. (2013), namely productive longevity (**PL**) (Brzáková et al., 2019), that uses the algorithm proposed by Jamrozik et al. (2013) to estimate genetic merit and transform it to an interpretable scale. In this context, PL was defined as the number of calves consecutively and regularly produced from the second to the eighth parity at 9 yr of age, assuming the first calf was produced at roughly 2 yr of age.

Longevity has been extensively investigated in purebred animals (Jamrozik et al., 2013; Silva et al., 2018; Oliveira et al., 2020). However, few studies have explored PL in beef cattle multibreed populations (Brzáková et al., 2019). With the use of crossbreeding to capture the benefits of maternal heterosis, the development of genetic evaluation programs that accommodate multiple breeds, genomic information, and evaluate economically important traits such as PL are essential to better inform decision making. Therefore, the purpose of this study was to develop a data screening strategy and a statistical model to predict genetic merit for PL in a multibreed beef cattle population.

## Materials and Methods

Animal Care and Use Committee approval was not obtained for this study, as the statistical analysis used historically collected data under standard farm management procedures.

### Data sources and the editing processes

Pedigree and calving date phenotype information was provided by Leachman Cattle of Colorado, Fort Collins, CO, that was retrieved from their multibreed performance recording program, which includes British, Continental, composite, as well as other breeds (i.e., dairy, Indicus, and Japanese breeds, among others). The distribution of purebred animals by breed group is included in Table 1. A total of 1,352,765 animals from 101,102 sires and 413,248 dams were available in the pedigree and used to obtain PL observations based on the algorithm described by Jamrozik et al. (2013). The editing process consisted of: 1) removing observations with missing information on dam, date of birth, and herd; 2) removing observations from cows having multiple births within a year, embryo transfer, and twins; 3) removing observations from cows that have their first calf with <18 mo and >36 mo; 4) removing observations from cows with calving intervals >730 d between two consecutive parities; 5) creating contemporary groups by combining dam’s herd and year-season of birth (January to June = season 1 and July to December = season 2) and removing contemporary groups with less than 3 cows, and with no variability for PL; 6) removing observations from cows born before 1970; and 7) removing observations from the first parity of all cows due to no variability, as each cow needs to calve roughly with 2 yr of age to be included in the dataset. After this process, the final dataset included 978,382 observations for PL on 160,748 cows born between 1970 and 2017 (calves born between 1972 and 2020). The algorithm proposed by Jamrozik et al. (2013) assumes that each cow in the dataset should calve every year, meaning that parity, cow age, and observation number are related (i.e., parity 2 = dam age 3 = observation 1, parity 3 = dam age 4 = observation 2, parity 4 = dam age 5 = observation 3, parity 5 = dam age 6 = observation 4, parity 6 = dam age 7 = observation 5, parity 7 = dam age 8 = observation 6, and parity 8 = dam age 9 = observation 7). Note that such a relationship might not hold for cows that had calving intervals between 365 and 730 d.

**Table 1.** Number of animals for the main breeds available in the Leachman Cattle of Colorado dataset used for PL research

Breeds	Number of animals
Black Angus	578,861
Red Angus	164,104
South Devon	38,840
Simmental	32,968
Gelbvieh	16,404
Charolais	9,894
Hereford	8,747
Brangus	5,133
Wagyu	2,762
Limousin	2,014
Others	11,552
Crossbreeds	481,486
Total	1,352,765

Genotypes for 26,342 animals were available and obtained from the Zoetis commercial genotyping laboratory. The samples were submitted to Zoetis for genomic testing; DNA was extracted, and genotyping was performed using Illumina BeadArray Single-Nucleotide Polymorphism (**SNP**) chips, with the number of markers ranging from about 40,000 to over 65,000. Genotypes were submitted to quality checks as described in Wiggans et al. (2009). Genotyping quality control consists of removing duplicate genotypes (>98% overlaps), animals and SNP with call rate <90%, genotypes with heterozygosity <20% and >80%, minor allele frequency <5%, and SNP with no mapped location in the genome. After genotyping quality control, the genotype file included 26,342 animals and 42,915 SNP.

### Statistical models and the estimation of variance components

#### Repeatability linear model

A repeatability linear model (REP) was developed for PL under a Bayesian inference framework as follows:


$$\begin{array}{l} y=Xb+Z_{u}u+Z_{p}p+Z_{c}c+e, \end{array}$$

where **y** was a binary vector of PL observations; **b** was a vector of the systematic effects including the year-season of calf’s date of birth, and the covariates of age at first calving, parity, pedigree-based retained heterosis which was computed as $RH=1-\;\left(\overset{n}{\underset{i=1}{\sum }}P_{si}P_{di}\right)$ where *P*_*si*_ and *P*_*di*_ are the breed composition fraction for the sire and dam relative to breed *i* obtained through pedigree information; **u** was the vector of additive genetic effects assumed to be $u∼N\left(0,A\sigma_{u}^{2}\right)$, where $\sigma_{u}^{2}$ is the additive genetic variance and **A** is the pedigree relationship matrix; **p** is the vector of permanent environmental effect with $p∼N\left(0,I\sigma_{p}^{2}\right)$ and $\sigma_{p}^{2}$ represent the permanent environmental variance; **c** was the vector of contemporary group effects with $c∼N\left(0,I\sigma_{c}^{2}\right)$ and $\sigma_{c}^{2}$ is the contemporary groups variance; **X**, **Z**_**u**_, **Z**_**p**_, and **Z**_**c**_ correspond to the incidence matrices for vectors **b**, **u**, **p**, and **c**, respectively; and **e** was the vector of residual effects distributed as $e∼N\left(0,I\sigma_{e}^{2}\right)$, where $\sigma_{e}^{2}$ was the residual variance; and **I** is an identity matrix with size relative to the associated random effect (i.e., permanent environmental, contemporary groups, and residual). The inclusion of the systematic effects in the model was assessed by biological importance and via statistical significance (*P* <0.05) by fitting a linear regression of each variable on PL at a time using the “*lm*” function available in the software *R* version 3.6.1 (R Core Team, 2021). Descriptive statistics of systematic effects are provided in Table 2. The distribution of PL was assumed as follows:

**Table 2.** Descriptive statistics for the systematic effects available for PL

Variables	Levels	Mean (SD)	Minimum	Maximum
Age at first calving (year)	–	2.02 (0.15)	1.50	2.67
Contemporary groups^1^	4,469	35.97	3	866
Pedigree retained heterosis	–	0.25 (0.35)	0	1
Year-season of birth	95	–	–	–

^1^Table entries represent the number of cows within the contemporary groups.


$$\begin{array}{l} y|b,u,c,p,\sigma_{e}^{2}\;∼\;N\left(Xb+Z_{u}u+Z_{p}p+Z_{c}c,I\sigma_{e}^{2}\right) \end{array}$$

in which a flat prior distribution was assigned to the systematic effects where b ∝constant; Gaussian distributions were used for the additive genetic, permanent environmental, and contemporary groups effects as $u|A,\sigma_{u}^{2}∼N\left(0,A\sigma_{u}^{2}\right)$, $p|A,\sigma_{p}^{2}∼N\left(0,I\sigma_{p}^{2}\right)$, and $c|A,\sigma_{c}^{2}∼N\left(0,I\sigma_{c}^{2}\right)$, respectively; and scaled inverted chi-square distributions were assumed for the variance components: $\sigma_{u}^{2}∼X_{u}^{-2}\left(v_{u},S_{u}\right)$, $\sigma_{p}^{2}∼X_{p}^{-2}\left(v_{p},S_{p}\right)$, $\sigma_{c}^{2}∼X_{c}^{-2}\left(v_{c},S_{c}\right)$, and $\sigma_{e}^{2}∼X_{e}^{-2}\left(v_{e},S_{e}\right)$ where v_*u*_, v_*p*_, v_*c*_, and v_*e*_ and S_*u*_, S_*p*_, S_*c*_, and S_*e*_ are the degrees of freedom and scale parameters for additive genetic, permanent environmental, contemporary group, and residual effects, respectively.

#### Random regression linear model

A linear random regression model (**RRM**) was also developed to investigate PL using Bayesian inference. In matrix notation, the RRM was defined as follows:


$$\begin{array}{l} y=Xb+Z_{u}u+Z_{p}p+Z_{c}c+e, \end{array}$$

where **y** was a binary vector of longevity observations, **b** was a vector of the systematic effects, **u** was the vector of the random regression coefficients for animal additive genetic effects, **p** was the vector of random regression coefficients for permanent environmental effects, **c** was the vector of the random regression coefficients for contemporary group effects, **X**, **Z**_**u**_, **Z**_**p**_, and **Z**_**c**_ correspond to the incidence matrices for vectors **b**, **u**, **p**, and **c**, respectively, and **e** was the vector of residual effects. The systematic effects included in **b** are year-season of calving and the covariates of age at first calving, parity fit as the average trajectory, and pedigree-based retained heterosis. For all random effects in the model, regression coefficients were regressed on parity to obtain the random regression coefficients.

RRM assumed that $y|b,u,p,c,G_{u},G_{p},G_{c},\sigma_{e}^{2}∼N(b+Z_{u}u$$+\;Z_{p}p+Z_{c}c,{I\sigma }_{e}^{2})$, in which $G_{u},G_{p},and\;G_{c}$ are the additive genetic, permanent environmental, and contemporary group (co)variance components matrices for the random regression coefficients, respectively; $\sigma_{e}^{2}$ is the residual variance with an inverted chi-squared distribution; **b**, **u**, **p**, and **c** were assumed to have flat distribution, $u|G_{u},H∼N\left(0,G_{u}⊗A\right)$, $p|G_{p}∼N\left(0,G_{p}⊗I_{p}\right)$, $c|G_{c}∼N\left(0,G_{c}⊗I_{c}\right)$ distributions, respectively, where **N** is a normal distribution function, **A** is the pedigree relationship matrix, **I**_**p**_ and **I**_**c**_ are the corresponding identity matrices for permanent environmental and contemporary group effects, respectively. $G_{u}$, $G_{p}$, and $G_{c}$ follow an Inverted Wishart distribution (**IW**) as $G_{u}∼IW\left(V_{u},n_{u}\right)$, $G_{u}∼IW\left(V_{u},n_{u}\right)$, and $G_{u}∼IW\left(V_{u},n_{u}\right)$ in which **V**_._ and n_._ are scale matrices and degrees of freedom related to the additive genetic, permanent environmental, and contemporary group effects, respectively.

#### Estimation of variance components

Variance components for PL were estimated for REP and RRM with *GIBBS3F90* version 1.83 available within the *BLUPF90* family (Misztal et al., 2018) using a pedigree traced back 5 generations from cows having records for PL and with phantom links and phantom parent groups (**PPG**) (Westell et al., 1988). PPG were composed of year of birth, sex and breed, and phantom links were created to connect all animals to purebred ancestral groups in which they were codified by combining year of birth, sex, and a unique identification. For simplicity, variance components were estimated with no genomic information, as previous studies have shown no relevant differences in variance components for models that included or disregard genetic markers (e.g., Parker Gaddis et al., 2014; Gonzalez-Peña et al., 2018). The Bayesian sampling process was performed for 100,000 iterations, where the initial 50,000 iterations were discarded as a burn-in period and sampled every 50 iterations. This sampling process resulted in 1,000 samples used for posterior statistical analyses. Convergence of the unknown parameters was monitored by visual inspection of the trace plots and by using the Geweke convergence criterion available in the *boa* (Bayesian Output Analysis) R package (Smith, 2007). For RRM, variance components for each parity were obtained by using the posterior mean of (co)variances of the random regression coefficients for contemporary groups, additive genetic, and permanent environmental effects as follows:


$$\begin{array}{l} \;\varphi \;=TG_{c}T,\;\;\;\vartheta =TG_{u}T,\;\;\;and\;\;\theta =TG_{p}T, \end{array}$$

where  φ ,   ϑ,   and  θ are the contemporary groups, additive genetic, and permanent environmental (co)variance matrices for PL, respectively, **T** is a 7 × 2 matrix containing a column of ones and one column with parity number (i.e., 2 to 8). $G_{c},\;G_{u},\;\;\;and\;\;\;G_{p}$ were previously defined and represent the (co)variance matrices of the random regression coefficients of contemporary group, additive genetic, and permanent environmental effects, respectively.

### Genomic evaluation

After the estimation of the variance components, estimated breeding values (**EBV**) were obtained with *blup90iod2* version 3.119 (Tsuruta et al., 2001), which uses iteration on data. For this analysis, genomic information was included in REP and RRM with the full pedigree and phantom parents and linking parents. All available information, including pedigree, genotypes and phenotypes, were evaluated simultaneously by using the single-step genomic BLUP methodology (ssGBLUP). In ssGBLUP, the inverse of the traditional pedigree relationship matrix, **A**^−1^, is replaced by the inverse of the **H** matrix that combines pedigree and genomic relationships (Legarra et al., 2009; Aguilar et al., 2010):


$$\begin{array}{l} H^{-1}=A^{-1}+\left[\begin{array}{lll} 0 & \;0\;0 & \;\left(\tau G^{-1}+\omega A_{22}^{-1}\right)\; \end{array}\right], \end{array}$$

where $A^{-1}$ is an inverse of the pedigree relationship matrix, $G^{-1}$ is an inverse of the genomic relationship matrix (VanRaden, 2008), $A_{22}^{-1}$ is an inverse of the pedigree relationship matrix including genotyped animals only, and τ and ω are scaling factors to condition the genomic relationship matrix to be compatible with the pedigree information in which both parameters were set to one. The PPG were accommodated in ssGBLUP via QP transformation (Misztal et al., 2013):


$$\begin{array}{l} H^{*}=A^{*}+\;\;\;\left[\begin{array}{lllllll} 0 & \;0 & \;0\;0 & \;G^{-1}+A_{22}^{-1} & \;-\left(G^{-1}+A_{22}^{-1}\right)Q_{2}\;0 & \;-Q_{}^{2^{\prime }}\left(G^{-1}+A_{22}^{-1}\right) & \;Q_{2}^{{\;}^{\prime }\;}\left(G^{-1}+A_{22}^{-1}\right)Q_{2}\; \end{array}\right], \end{array}$$

where $H^{*}$ and $A^{*}$ are $H^{-1}$ and $A^{-1}$ modified relationship matrices based on QP transformation (Quaas, 1988), respectively, and $Q_{2}$ is a matrix assigning genotyped animals to PPG. For this analysis, inbreeding was considered when constructing the pedigree relationship matrix and model convergence was achieved at a threshold of 10^-18^ using the option *avgeps* with 50 iterations to ensure stable solutions.

### Expressing EBV as number of calves

Similar to Oliveira et al. (2020), EBV was transformed into a number of calves by fitting a linear regression of the EBV on the number of calves produced per cow. To ensure that each cow had the opportunity to produce up to eight calves, a subset of females born from 1970 to 2010 were used in the transformation. A simple linear regression was performed on this subset, and then EBV was expressed as follows:


$$\begin{array}{l} {\bar{EBV}}_{NC}=b_{0}+b_{1}\bar{EBV}, \end{array}$$

where ${\bar{EBV}}_{NC}$ is the EBV expressed as the number of calves produced up to eighth parity, $\bar{EBV}$ is the EBV obtained from REP or RRM, and b_0_ and b_1_ are the intercept and the regression coefficient related to $\bar{EBV}$. For RRM, ${\bar{EBV}}_{NC}$ was computed considering the $\bar{EBV}$ computed at the eighth parity.

### Validation approach

The predictive performance for ${\bar{EBV}}_{NC}$ was assessed via a modified forward validation approach where a group of animals have their EBV results followed over the years. In this procedure, the main idea is to mimic the evolution of the genetic evaluation by excluding data that were not available upon a threshold year and comparing its results with the most recent genetic evaluation. In our study, a group of 7,268 cows born in 2010 were selected and followed throughout the years as they accumulated phenotypic calving records. The selection of these cows was made based on the youngest group of animals in the dataset that had the opportunity to produce 8 calves, avoiding the use of cows with censored records. The oldest dataset was generated by removing the phenotypic, genomic, and pedigree information for all animals born after December 31, 2010. The next dataset removed animals born after December 31, 2013, where those animals born in 2010 had their first measure for PL. Subsequently, the same concept was extended for the years 2014 to 2019 until we had the current dataset used for the genetic evaluation. The total number of calves produced per cow were precorrected for nongenetic effects by using *predictf90* program version 1.3 (Misztal et al., 2018). Pearson’s correlations between the ${\bar{EBV}}_{NC}$ obtained from the reduced datasets (e.g., 2010, 2013 to 2019) and the observed and precorrected total number of calves produced per cow was computed considering the 7,268 cows available in the validation set. In addition, bias was assessed by applying a linear regression between ${\bar{EBV}}_{NC}$ obtained in each year and those computed in 2020.

### Statistical models comparison

The REP and RRM were compared based on computational efficiency and similarity between EBV solutions. To assess the first criterion, the number of iterations and time to reach convergence in the *blup90iod2* program were considered, while for the second criterion, Spearman correlation between EBV solutions obtained from REP and RRM were computed and the percentage of animals that were selected in common considering the top 1% of individuals.

## Results and Discussion

**Figure 1.**
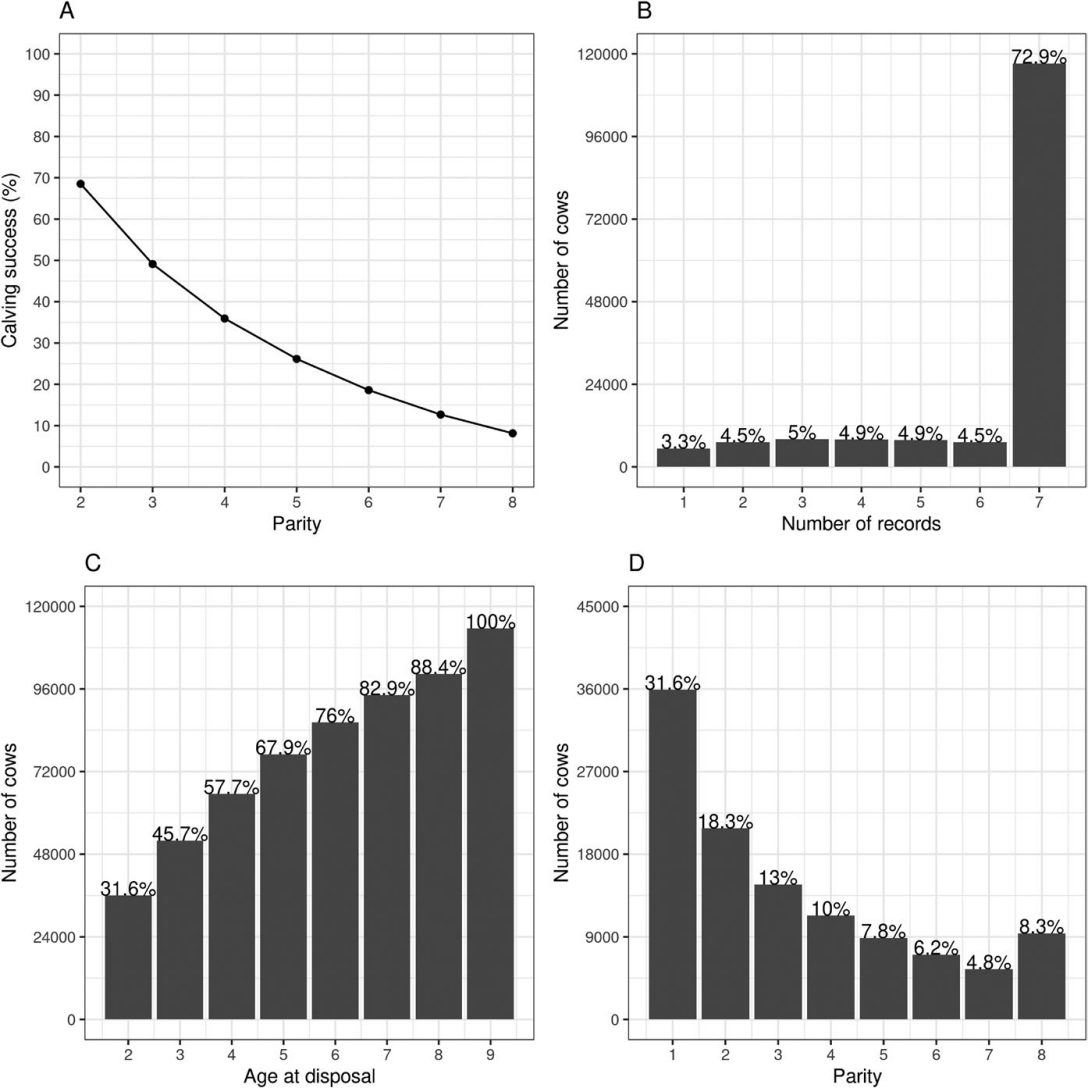
Descriptive statistics of (**A**) average calving success rate described by parity, (**B**) distribution of number of records by number of cows, (**C**) number of cows by age at disposal, and (**D**) number of cows by number of calves.

**Figure 2.**
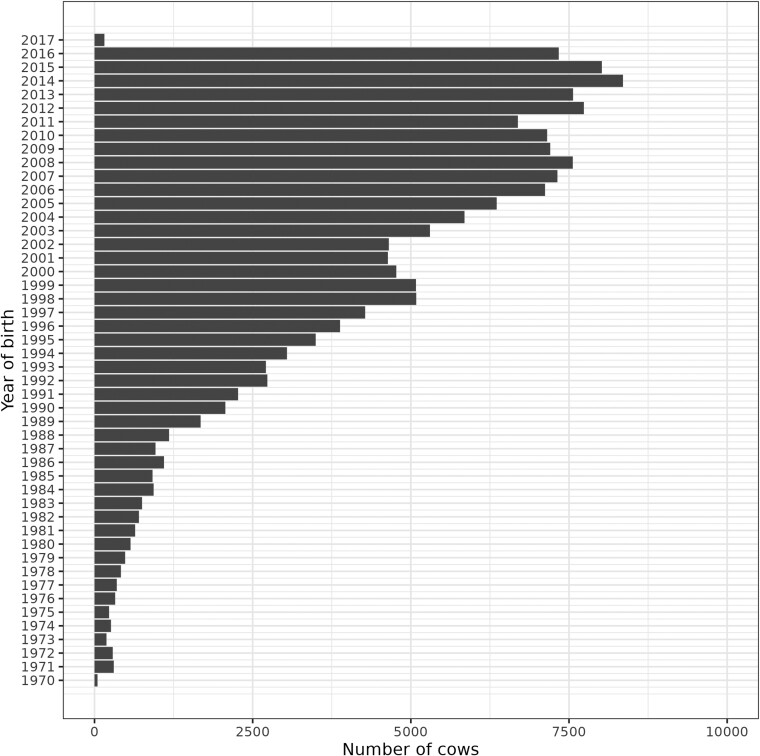
Number of cows by year of birth.

Overall, the calving success rate decreased as the number of parities increased, where 68% and 8% of all cows in the data produced calves at the second and eighth parities, respectively (Figure 1A). A similar finding was reported by Jamrozik et al. (2013) and Silva et al. (2018), who showed ~70% and ~10% calving success rates in Simmental and Zebu beef cattle at the second and eight parities, respectively. Approximately 73% of cows had 7 PL observations, and the remainder (27%) had up to 6 observations for PL (Figure 1B). This result highlights that about 73% of the cows were old enough to have the opportunity to reach eight parities given that they calved or not (Figure 2), while the remaining 27% had censored records as they were born after 2010. Overall, the number of cows increased over the years as genetic evaluation grew with the enrollment of new partners and improved data recording. Few cows were integrated into the database in 2017 due to the censored nature of the data in which statistical analyses were performed before cows born in 2017 could calve.

Most of the cows in the data (82.9%) were disposed from the herd before reaching 7 yr of age (Figure 1C). This value was comparable to that reported by Oliveira et al. (2020), who found that 82% of the Angus cows in the United States left the herd before 10 yr of age. The observed difference of 3 yr may reflect the range of parities considered in each study (8 parities vs. 15 parities). In our study, 31.6% of the cows calved once in their life, while 8.3% had 8 calves (Figure 1D). These results roughly align with the literature reports in which Brzáková et al. (2019) reported that 22% of the beef cattle cows from the Czech Republic have only one calf, and Oliveira et al. (2020) communicated that 20.6% of the Angus cows from the United States calved just once in their life.

**Figure 3.**
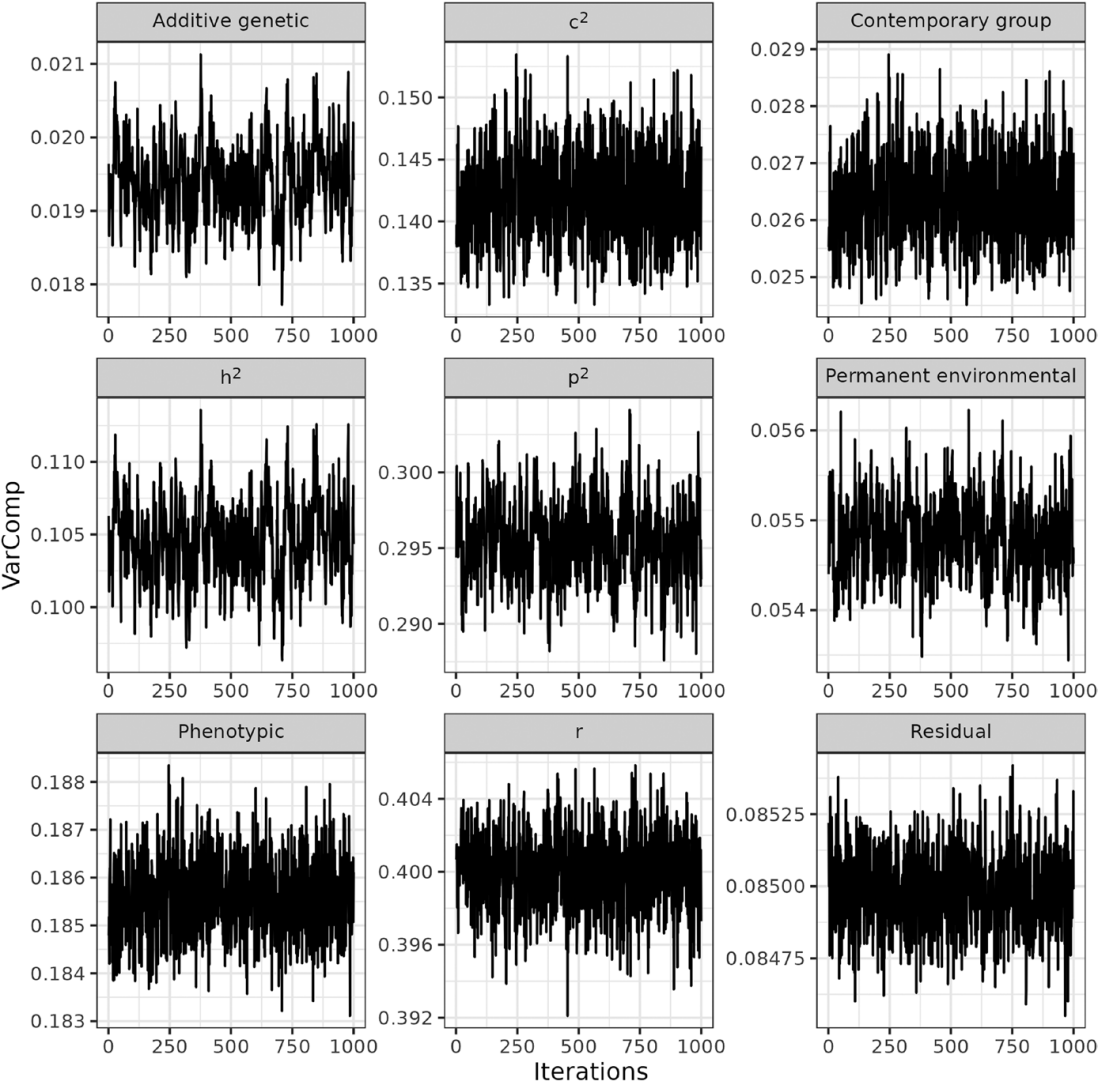
Trace plots for the additive genetic variance, permanent environmental variance, contemporary groups variance, residual variance, phenotypic variance, heritability (*h*^2^), the ratio between permanent environmental and phenotypic variances (*p*^2^), and the ratio between the contemporary group and phenotypic variances (*c*^2^), and the repeatability (*r*) obtained with the repeatability model.

**Figure 4.**
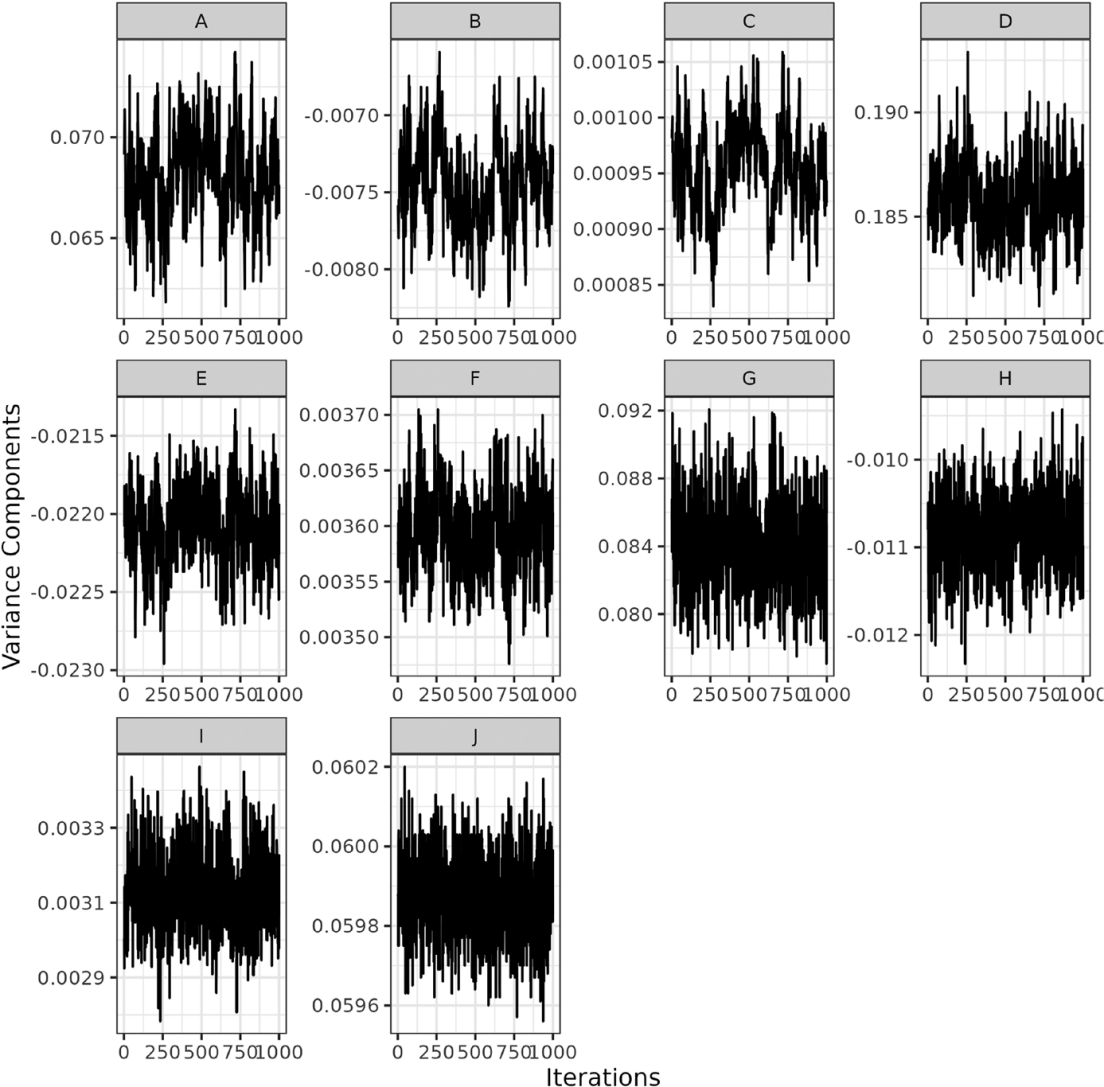
Trace plot of the regression coefficient variance components of the RRM for (A) intercept additive genetic, (B) covariance between intercept and slope additive genetic, (C) slope additive genetic, (D) intercept permanent environmental, (E) covariance between intercept and slope permanent environmental, (F) slope permanent environmental, (G) intercept contemporary groups, (H) covariance between intercept and slope contemporary groups, (I) slope contemporary groups, and (J) residual variance.

**Figure 5.**
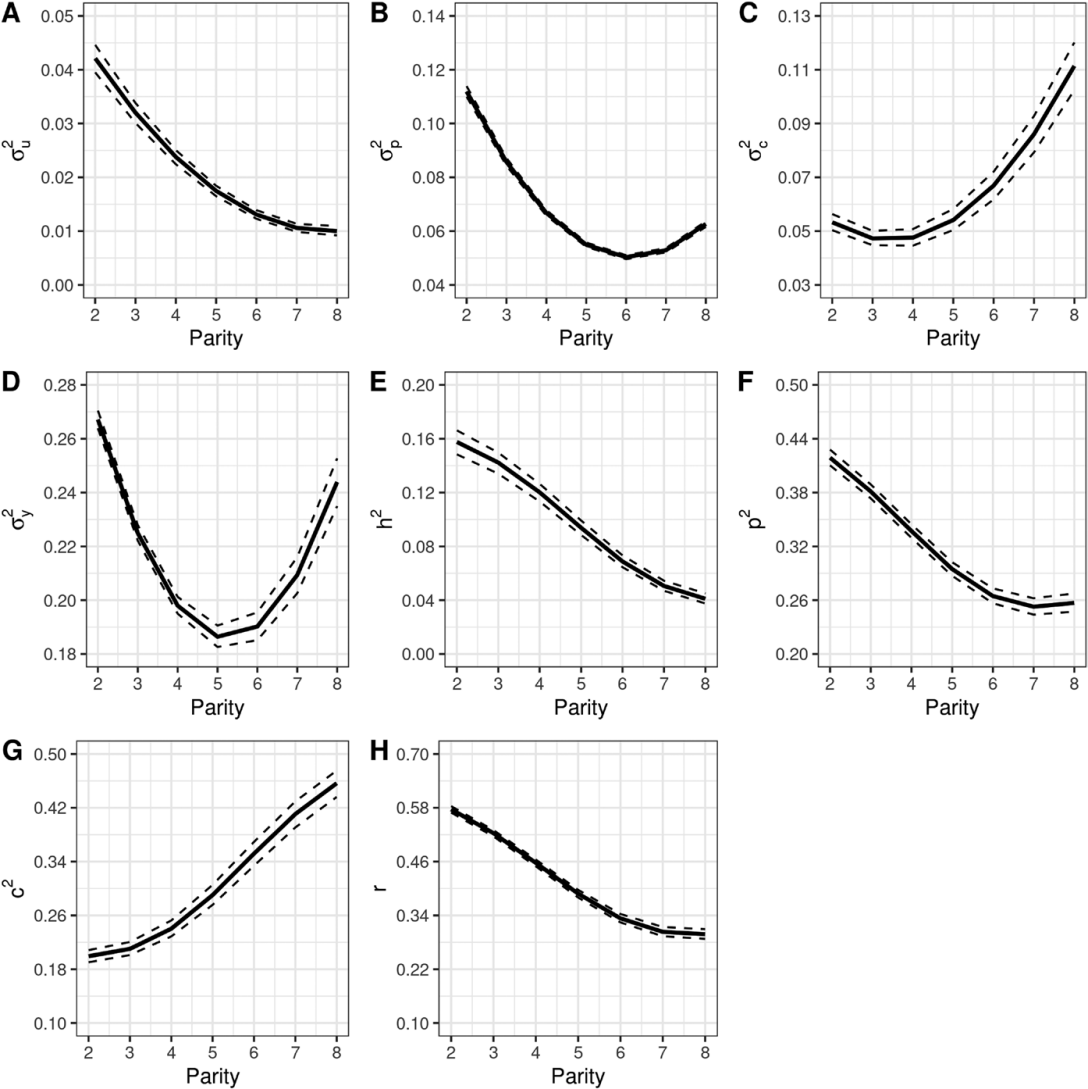
Variance components and genetic parameters of the RRM are described by calving where (A) additive genetic, (B) permanent environmental, (C) contemporary groups, (D) phenotypic, (E) heritability, (F) ratio between permanent environmental and phenotypic, and (G) ratio between contemporary group and phenotypic.

Posterior means (µ), 95% posterior highest density intervals (HPD_95%_), and trace plots of the variance components for REP and RRM are presented in Tables 3 and 4 and Figures 3 and 4, respectively. All variance components reached the stationary state based on trace plots and the Geweke criterion (*P* > 0.05) for REP and RRM. Such results suggest that the variance components converged, and statistical inference was appropriate for these parameters and models. Calus et al. (2016) reported that a Gibbs chain based on 100,000 iterations and a burn-in of 50,000 iterations were sufficient to estimate unknown parameters in their genomic prediction study. For REP, the variance of the residual effects accounted for most of the phenotypic variation (Table 3, µ: 0.081; HPD_95%_: 0.080 to 0.082), followed by the variances of the permanent environment (Table 3, µ: 0.056; HPD_95%_: 0.056 to 0.058), contemporary group (Table 3, µ: 0.026; HPD_95%_: 0.025 to 0.028), and additive genetic effects (Table 3, µ: 0.019; HPD_95%_: 0.018 to 0.020). For RRM, the phenotypic and permanent environmental variance curves displayed a parabolic shape with minimum values at the fifth and sixth parities, respectively (Figure 5D and B). The additive genetic variance curve presented a decreasing trend from the second to the eighth parity (Figure 5A), while contemporary group variances were almost constant up to the fourth parity with an increasing trend afterwards (Figure 5C). Overall, the trends of the variance components in RRMs are often different depending on the studies (Jamrozik et al., 2013; Silva et al., 2018), reflecting differences in the dataset used and the type and order of the polynomial considered to adjust longitudinal data (e.g., natural vs. Legendre, and linear vs. third/fourth order). Heritability estimates were, on average, 0.107 (HPD_95%_: 0.102 to 0.112) for REP (Table 3) and showed a decreasing trend for RRM, ranging from 0.16 to 0.04 (Figure 5E). These results align with those found by Brzáková et al. (2019), who reported heritability estimates below 0.14 for PL defined as the number of calves produced up to four ages (e.g., 78, 90 150, and 160 mo) in a multibreed population using single-trait and multiple-trait models. Similar heritability estimates were also reported by Oliveira et al. (2020), whose values ranged from 0.07 to 0.25 for traditional longevity and from 0.07 to 0.25 for functional longevity in an Angus population from the United States. On the other hand, heritability estimates were lower than those reported by Jamrozik et al. (2013), who investigated stayability to consecutive calvings and found estimates ranging from 0.13 to 0.35 in a Simmental population from Canada. Higher heritability estimates for stayability (ranging from 0.18 to 0.25) were also observed in a Hereford population (Martinez et al., 2005). Although heritability estimates found in our study were relatively low in magnitude and show a relevant role of the environmental factors in the expression of the phenotype, selecting for PL is feasible and can potentially contribute to genetic progress over time. Conversely, repeatability was of moderate magnitude 0.415 (HPD_95%_: 0.410 to 419) for REP (Table 3) and showed a decreasing trend for RRM with values ranging from 0.57 to 0.29 (Figure 5H). These estimates are lower than those found by Jamrozik et al. (2013), which ranged from 0.61 to 0.91 in Canadian Simmental cattle. The repeatability estimates for this study indicate that the first observation of PL may be sufficient to provide a good prediction of subsequent performance. The ratio of the contemporary group variance to the phenotypic variance was 0.142 (HPD_95%_: 0.134 to 150) for REP, and it showed an increasing trend for RRM with values ranging from 0.199 to 0.456. In this study, contemporary groups were treated as a random effect due to convergence issues on the variance components when assuming such effect as systematic. Jamrozik et al. (2013) reported approximately constant estimation for the ratio between contemporary group and phenotypic variances, while Silva et al. (2018) found a decreasing trend through parities. Such differences in trends may reflect heterogeneous herd management across farms, such as the environment where cows were raised (e.g., confinement vs. pasture), type of matching (natural vs. artificial insemination), nutritional protocols (managing first-calf heifers separately), and specific culling decisions.

**Table 3.** The posterior mean, the 95% highest posterior density interval (HPD_95%_), and the Geweke diagnostic *P*-value for the variance components estimated with the repeatability model

Variance components	Mean	HPD_95%_	Geweke *P*-value
$\sigma_{a}^{2}$	0.019	0.018 to 0.020	0.215
$\sigma_{p}^{2}$	0.057	0.056 to 0.058	0.105
$\sigma_{c}^{2}$	0.026	0.025 to 0.028	0.614
$\sigma_{e}^{2}$	0.081	0.080 to 0.082	0.355
$\sigma_{y}^{2}$	0.185	0.183 to 0.186	0.539
*h* ^2^	0.107	0.102 to 0.112	0.233
*p* ^2^	0.307	0.303 to 0.313	0.094
*c* ^2^	0.142	0.135 to 0.149	0.641
*r*	0.415	0.410 to 0.419	0.957

$\sigma_{.}^{2}$ is the variance component of additive genetic (*a*), permanent environmental (*p*), contemporary groups (*c*) or residual effect (*e*).

$h^{2}$ is the heritability of PL.

$p^{2}$ is the ratio between permanent environment and phenotypic variances.

$c^{2}$ is the ratio between contemporary group and phenotypic variances.

*r* is the repeatability of PL.

**Table 4.** The posterior mean, the 95% highest posterior density interval (HPD_95%_), and the Geweke diagnostic *P*-value for the variance components of the regression coefficients of the RRM

Variance components	Mean	HPD_95%_	Geweke p-value
$\sigma_{a_{1}}^{2}$	0.068	0.063 to 0.0072	0.442
$\sigma_{a_{1}a_{2}}$	−0.007	−0.008 to −0.006	0.918
$\sigma_{a_{2}}^{2}$	0.0009	0.0008 to 0.0010	0.346
$\sigma_{p_{1}}^{2}$	0.185	0.182 to 0.189	0.849
$\sigma_{p_{1}p_{2}}$	−0.023	−0.024 to −0.022	0.710
$\sigma_{p_{2}}^{2}$	0.0035	0.0034 to 0.0036	0.177
$\sigma_{c_{1}}^{2}$	0.084	0.078 to 0.088	0.13
$\sigma_{c_{1}c_{2}}$	−0.010	−0.012 to −0.009	0.101
$\sigma_{c_{2}}^{2}$	0.0031	0.0028 to 0.0033	0.839
$\sigma_{e}^{2}$	0.059	0.058 to 0.060	0.421

$\sigma_{._{1}}^{2}$ is the variance of the intercept of the additive genetic (*a*), permanent environmental (*p*) or contemporary groups (*c*).

$\sigma_{._{1}._{2}}$ is the covariance between the intercept and slope of the additive genetic (*a*), permanent environmental (*p*), and contemporary groups (*c*).

$\sigma_{._{2}}^{2}$ is the variance for the slope of the additive genetic (*a*), permanent environmental (*p*) or contemporary groups (*c*).

$\sigma_{e}^{2}$ is the residual variance.

Repeatability models assume that the genetic correlation of repeated measurements is equal to one across time, meaning that the set of genes which control a trait is the same during the evaluated period. Such an assumption may not reflect reality because the sets of genes that control many complex traits could change over time leading to genetic correlations lower than one. Genetic correlations among parities ranged between 0.43 to 0.99 for RRM. This result is aligned with the literature reports in which genetic correlations for longevity were of moderate to high magnitude, ranging from 0.57 to 0.96 for different parities (Jamrozik et al., 2013; Silva et al., 2018). These results indicate that RRM could better accommodate changes in genetic correlations over time than REP. Although RRM is theoretically preferred to study longitudinal data where a trait changes gradually and continually over time (Meyer, 2004, 2005; Schaeffer, 2004), we found that implementing REP in a large commercial genetic evaluation program reduces complexity and provides comparable results to RRM. In our study, the solutions of RRM converged with ~4 times more iterations than REP (3,583 vs. 845) and took 53 min to reach convergence against 7 min for REP. Moreover, the Spearman correlation between EBV solutions obtained by RRM and REP was 0.96 and 77% of the animals (9,821 out 12,746) ranked in the top 1% were selected in common in both models. These results indicate that RRM requires more time and iterations to reach convergence than REP to yield similar solutions. These findings could be partially explained by the higher number of parameters and complexity of the RRM compared to REP. It is important to notice that in general, the amount of data in commercial genetic evaluations will continue to grow, which subsequently could make the use of RRM troublesome. Therefore, to have a stable commercial genetic evaluation for PL it is recommended to use REP based on the available data. Henceforth, all subsequent results reported in this study are from REP.

**Figure 6.**
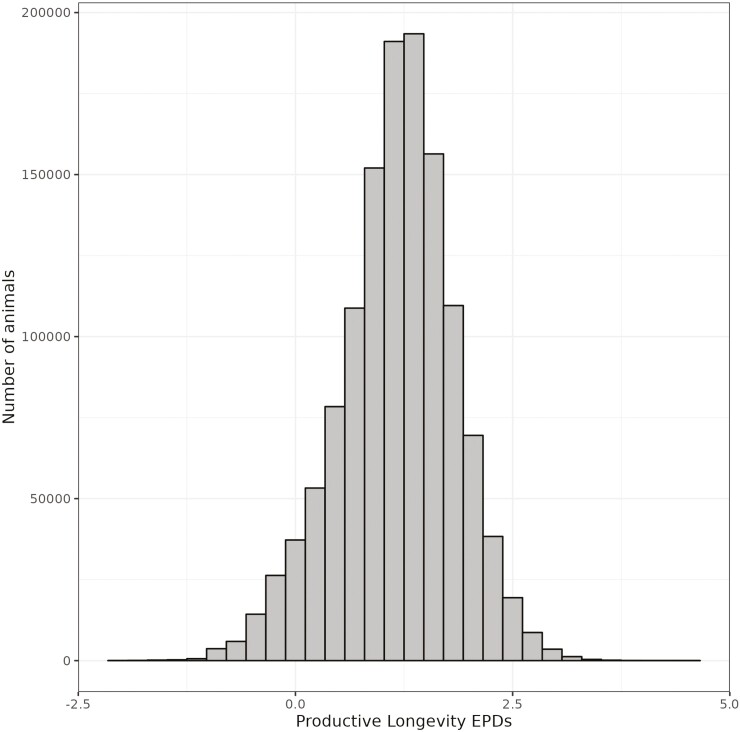
Histogram distribution of the EPD transformed to the number of calves for PL.

**Figure 7.**
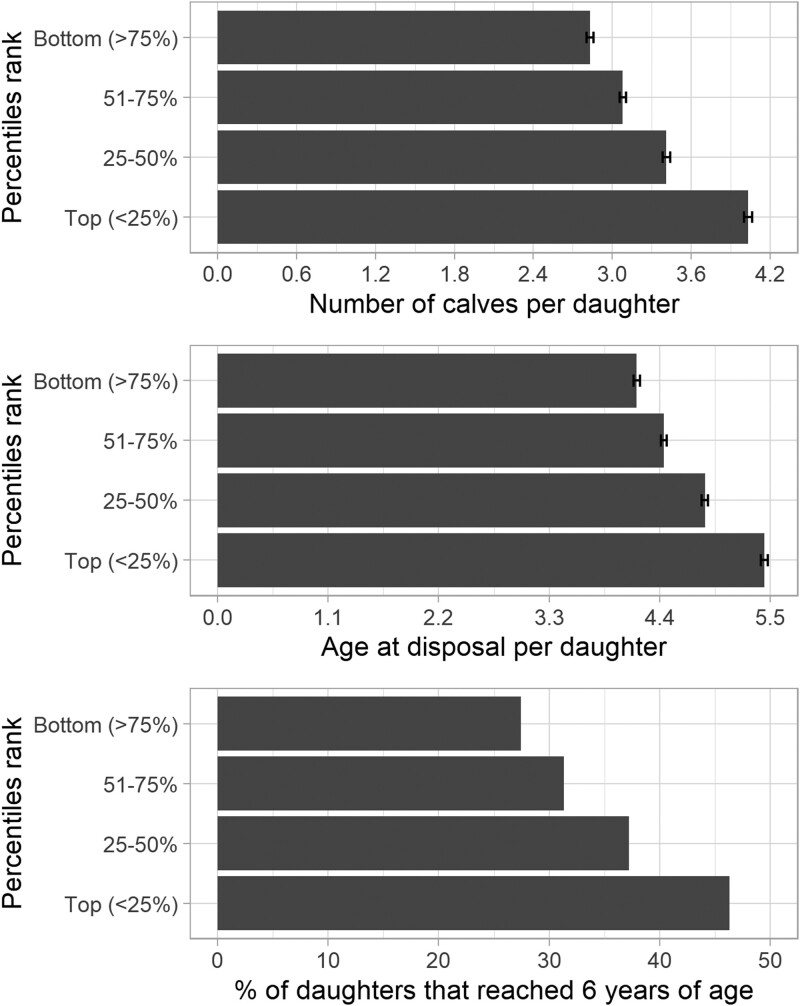
The average performance of daughters born before 2010 from 109 bulls with ≥0.70 Beef Improvement Federation accuracy ranked based on their EPD. Plots represent the average performance of the daughters in number of calves produced (top), age at disposal (middle), and percentage of daughters that reached 6 yr of age (bottom).

The distribution of expected progeny difference (**EPD**) for PL ranged from −2.03 to 4.55, representing a considerable difference of 6.58 calves when comparing the best and the worst individuals in the database (Figure 6). Moreover, when investigating the performance of the daughters born before 2010 from 109 bulls with ≥0.70 Beef Improvement Federation (**BIF**) accuracy, that ranked in the top (≤25%), 26 to 50%, 51 to 74%, and bottom (≥75%) quartiles based on their EPDs, we observed aligned differences in their daughters’ performance in number of calves produced, age at disposal, and percentage of daughters that reached 6 yr of age (Figure 7). The average number of calves produced per daughter aligned and increased across the quartile PL genetic groups, with a difference of 1.20 calves between the top and bottom groups. A similar trend was observed for age at disposal and the percentage of daughters that reached 6 yr of age, with a difference between the top and bottom groups of 1.27 yr and 18.87%, respectively. These results suggest that choosing sires with high genetic merit for PL could help optimize their use, leading to more productive cows and improved profit for cow-calf producers.

For the modified forward validation approach, Pearson’s correlations between EBV and precorrected phenotypes and EBV and observed phenotypes increased over the years, being high for datasets that used the most recent records, ranging from 0.44 to 0.92 and from 0.04 to 0.66, respectively (Table 5). Such a trend was expected as the amount of data flowing into the database increased throughout the years, growing to the most recently available data. Correlations were 0.44 between EBV and precorrected phenotypes in 2010, when animals have only the pedigree and/or genotype information. Such correlation was relatively high considering the low trait heritability and given that cows in the validation dataset had only pedigree and/or genomic information. Therefore, this result suggests that the ${\bar{EBV}}_{NC}$ is a good indicator associated with future cow performance measured in the total number of calves. Another interesting outcome is that the correlation between the precorrected phenotypes and ${\bar{EBV}}_{NC}$ increased almost 50% in 2013, as well as the correlation between observed phenotypes and ${\bar{EBV}}_{NC}$ improved from 0.04 to 0.44, suggesting that the inclusion of the first phenotypic record (second parity output: success or failure) in the validation set is moderately related to their final productive performance. Bias estimates were <1 for each dataset available in the validation, increasing from 0.64 (2010) to 0.99 (2019). Such a result indicates an inflation of the EBV, meaning that EBV estimates were larger in absolute value than expected. As more data has accumulated over the years, EBV has become less biased and closer to the most recent EBV computed in 2020.

**Table 5.** Bias and Pearson’s correlations between the transformed EPD and precorrected/observed total number of calves produced per cow for 131 cows born in 2010

		Pearson’s correlations	
Year	Bias	Precorrected	Observed
2010	0.64	0.44	0.04
2013	0.89	0.67	0.42
2014	0.92	0.77	0.56
2015	0.94	0.83	0.64
2016	0.95	0.86	0.67
2017	0.98	0.91	0.67
2018	0.98	0.92	0.66
2019	0.99	0.92	0.65

Incorporating PL into breeding programs is fundamental for increasing net returns of cow-calf producers by having longer-lived, more productive cow inventories and lower associated rates of heifer retention. Specifically, with the implementation and use of PL EPD proposed in this study, it is possible to identify sires, as well as candidate replacement heifers (daughters), with genetic potential to regularly produce more calves, leading to lower cow depreciation costs. Historically, genetic selection for PL has been challenging because of its low heritability, length of time required for lifelong expression of phenotypes, and the lack of recorded culling reasons. However, with the advance of genotyping technologies and use of inventory-based data management systems, selecting for PL is feasible. Such innovation enables the reduction of generation interval and with improved data recording at the farm and animal levels, enhanced accuracy of selection for young animals.

To the best of the authors’ knowledge, no other study investigated PL using genomic information in a single-step evaluation of a large multibreed beef cattle population. Including purebred and crossbred data simultaneously in a combined multibreed genomic evaluation could improve accuracies of predictions for all animals. Winkelman et al. (2015) reported a gain in predictive accuracy for purebred and crosses when considering data from Holsteins, Jerseys, and crosses in New Zealand. Similarly, Khansefid et al. (2020) working with a population of Holsteins, Jerseys, and crosses from New Zealand and the Netherlands reported gains in the accuracy of prediction when mixing purebred and crossbred data in the reference population. Despite such benefits, multibreed genomic evaluations are challenging to implement due to differences in population structure among breeds (i.e., allele frequencies, linkage disequilibrium, and effective population size), which may impact model assumptions and EBV estimates, especially for breeds that are not well represented in the reference population. Multibreed genomic evaluation is a topic that requires further investigation, and it is crucial to have additional studies to understand PL in such populations.

## Conclusions

In this study, we used a repeatability model to evaluate an alternative definition of PL in a multibreed population, in which PL was defined as the number of calves consecutively and regularly produced from the second to the eighth parity at 9 yr of age, assuming the first calf was produced at roughly 2 yr of age. Heritability was of relatively low magnitude, indicating that most of the phenotypic variation is accounted for by environmental effects, but selecting for PL is still possible and could potentially contribute to the genetic progress over time. Repeatability estimates and the validation method suggested that the first measurement of the trait (second parity calving) is an effective indicator of posterior performance. Therefore, based on favorable evaluations of efficacy, the inclusion of PL in a multibreed genetic evaluation program, and through PL EPD and inclusion in selection indexes with existing economic traits, could yield more profitable selection and breeding decisions.

## Data Availability

All data used in this study are from a proprietary genetic evaluation and cannot be made publicly available. Certain data might be available for research purposes under request by contacting Leachman Cattle of Colorado.

## Abbreviations

BIF: Beef Improvement Federation
EBV: estimated breeding value
EBV_NC_: estimated breeding value transformed to number of calves
EPD: expected progeny difference
HPD_95%_: , 95% posterior highest density interval
PL: productive longevity
PPG: phantom parent groups
REP: repeatability model
RRM: random regression model
SNP: single-nucleotide polymorphism
ssGBLUP: single-step genomic best linear unbiased predictor
